# Phenome-wide association of 1809 phenotypes and COVID-19 disease progression in the Veterans Health Administration Million Veteran Program

**DOI:** 10.1371/journal.pone.0251651

**Published:** 2021-05-13

**Authors:** Rebecca J. Song, Yuk-Lam Ho, Petra Schubert, Yojin Park, Daniel Posner, Emily M. Lord, Lauren Costa, Hanna Gerlovin, Katherine E. Kurgansky, Tori Anglin-Foote, Scott DuVall, Jennifer E. Huffman, Saiju Pyarajan, Jean C. Beckham, Kyong-Mi Chang, Katherine P. Liao, Luc Djousse, David R. Gagnon, Stacey B. Whitbourne, Rachel Ramoni, Sumitra Muralidhar, Philip S. Tsao, Christopher J. O’Donnell, John Michael Gaziano, Juan P. Casas, Kelly Cho, on behalf of the VA Million Veteran Program COVID-19 Science Initiative

**Affiliations:** 1 Massachusetts Veterans Epidemiology Research and Information Center (MAVERIC), VA Boston Healthcare System, Boston, Massachusetts, United States of America; 2 Department of Epidemiology, Boston University School of Public Health, Boston, Massachusetts, United States of America; 3 VA Salt Lake City Health Care System, Salt Lake City, Utah, United States of America; 4 Office of Research and Development, Veterans Health Administration, Washington, DC, United States of America; 5 Department of Medicine, University of Utah School of Medicine, Salt Lake City, Utah, United States of America; 6 Department of Medicine, Division of Aging, Brigham & Women’s Hospital, Boston, Massachusetts, United States of America; 7 Department of Medicine, Harvard Medical School, Boston, Massachusetts, United States of America; 8 Durham VA Medical Center, Durham, North Carolina, United States of America; 9 Department of Psychiatry and Behavioral Sciences, University Medical Center, Durham, North Carolina, United States of America; 10 VA Mid-Atlantic Mental Illness Research Education and Clinical Center, Durham, North Carolina, United States of America; 11 Corporal Michael Crescenz VA Medical Center, Philadelphia, Pennsylvania, United States of America; 12 Department of Medicine, Perelman School of Medicine, University of Pennsylvania, Philadelphia, Pennsylvania, United States of America; 13 Department of Biomedical Informatics, Harvard Medical School, Boston, Massachusetts, United States of America; 14 Department of Biostatistics, Boston University School of Public Health, Boston, Massachusetts, United States of America; 15 Department of Medicine, Stanford University School of Medicine, Stanford, California, United States of America; 16 VA Palo Alto Health Care System, Palo Alto, California, United States of America; Emory University, UNITED STATES

## Abstract

**Background:**

The risk factors associated with the stages of Coronavirus Disease-2019 (COVID-19) disease progression are not well known. We aim to identify risk factors specific to each state of COVID-19 progression from SARS-CoV-2 infection through death.

**Methods and results:**

We included 648,202 participants from the Veteran Affairs Million Veteran Program (2011-). We identified characteristics and 1,809 ICD code-based phenotypes from the electronic health record. We used logistic regression to examine the association of age, sex, body mass index (BMI), race, and prevalent phenotypes to the stages of COVID-19 disease progression: infection, hospitalization, intensive care unit (ICU) admission, and 30-day mortality (separate models for each). Models were adjusted for age, sex, race, ethnicity, number of visit months and ICD codes, state infection rate and controlled for multiple testing using false discovery rate (≤0.1). As of August 10, 2020, 5,929 individuals were SARS-CoV-2 positive and among those, 1,463 (25%) were hospitalized, 579 (10%) were in ICU, and 398 (7%) died. We observed a lower risk in women vs. men for ICU and mortality (Odds Ratio (95% CI): 0.48 (0.30–0.76) and 0.59 (0.31–1.15), respectively) and a higher risk in Black vs. Other race patients for hospitalization and ICU (OR (95%CI): 1.53 (1.32–1.77) and 1.63 (1.32–2.02), respectively). We observed an increased risk of all COVID-19 disease states with older age and BMI ≥35 vs. 20–24 kg/m^2^. Renal failure, respiratory failure, morbid obesity, acid-base balance disorder, white blood cell diseases, hydronephrosis and bacterial infections were associated with an increased risk of ICU admissions; sepsis, chronic skin ulcers, acid-base balance disorder and acidosis were associated with mortality.

**Conclusions:**

Older age, higher BMI, males and patients with a history of respiratory, kidney, bacterial or metabolic comorbidities experienced greater COVID-19 severity. Future studies to investigate the underlying mechanisms associated with these phenotype clusters and COVID-19 are warranted.

## Introduction

The burden of the novel coronavirus (SARS-CoV-2) in the United States (US) has been unprecedented, with the highest number of confirmed cases and deaths in the world [1–7]. It is now clear that substantial variability in the presentation of COVID-19 exists, ranging from asymptomatic or mild-symptoms to severe complications such as acute respiratory distress syndrome or multi-organ failure.

Risk factors for severe COVID-19 and death include male sex, older age, lower socioeconomic status, cardiovascular disease, type-2 diabetes mellitus (T2DM), asthma [7–26]. With a few exceptions, the large majority of current findings are from hospital-based studies that may not represent the broader at-risk population [27, 28]. Additionally, the evidence on risk factors is fragmented, with studies to date focusing on single outcomes rather than covering the progression of COVID-19 from diagnosis through hospitalization, ICU admission, and death. There is still a need to examine risk factors across all severity levels of disease, in order to differentiate risk factors associated with asymptomatic or mild cases from other risk factors associated with hospitalization and death.

To address this gap in knowledge, we leverage the Million Veteran Program (MVP) cohort, a longitudinal mega biobank with on-going recruitment of Veterans who receive care from the Veterans Health Administration (VA) and has contributed to numerous biomedical and genomics studies [29–31]. The VA is the largest single-payer healthcare system in the US and has over 20 years of electronic health record (EHR) data for 6 million annual active users nationwide which will allow to examine the history of clinical risk factors and comorbidities that may be associated with COVID-19.

The primary aims of the present investigation are to characterize the progression of COVID-19 from diagnostic testing through SARS CoV-2 infection, and outcomes after infection including hospitalization, ICU admission, and death; and to identify risk factors specific to each state of COVID-19 disease progression. To this end, we evaluated the association between 1809 phenotypes across major disease domains [32] with each stage of COVID-19 disease progression.

## Material and methods

### Study sample

**Fig 1:**
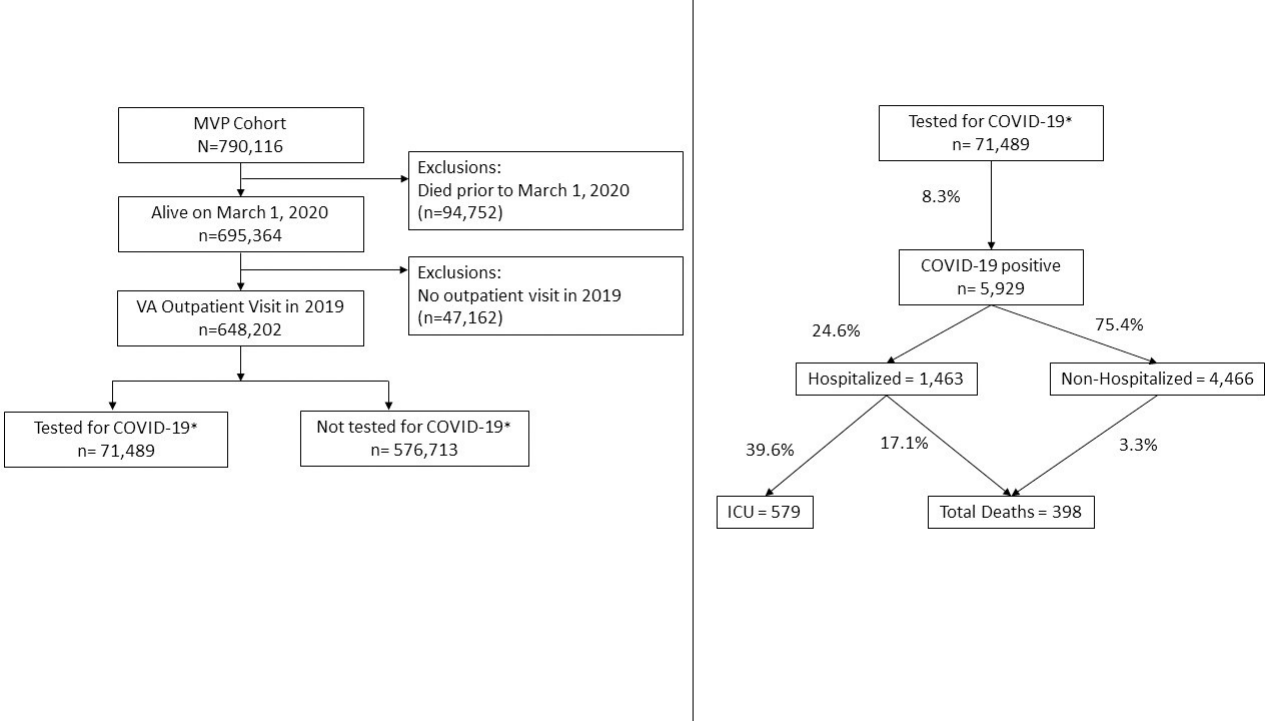
STROBE diagram of study sample. Flow diagram of Million Veteran Program participants included and excluded in each analysis, and number of participants who were SARS CoV-2 positive, hospitalized, admitted to the ICU, or died.

We included individuals enrolled in MVP, an ongoing longitudinal study that began in 2011 and was designed to study genetic and non-genetic determinants of diseases among U.S. Veterans [33]. The STROBE diagram in Fig 1 describes the inclusion criteria for MVP participants who are active VA users in the current analysis.

Briefly, as of November 13, 2019, there were 790,116 Veterans enrolled in MVP. We excluded 94,572 participants who died before March 1, 2020, before COVID-19 testing began in the VA, and 47,162 who did not have a VA clinic visit in the 2019 calendar year. Among the 648,202 remaining individuals, 71,489 (11%) were tested for SARS-CoV-2, of which 5,929 (8.3%) were tested positive from March 1, 2020 to August 10, 2020. Each MVP participant provided written informed consent, and the VA Central Institutional Review Board (IRB) approved the study protocol. MVP abides by a coded data standard and the data used in these analyses are void of participant names and other identifiable information. However, a unique ID code is assigned and used for the duration of the study activities.

### COVID-19 case and disease progression definition

COVID-19 cases were identified using an algorithm developed by the VA COVID National Surveillance Tool (NST) [34]. The NST classified COVID-19 cases as positive and negative based on reverse transcription polymerase chain reaction (RT-PCR) laboratory test results conducted at VA clinics, supplemented with Natural Language Processing (NLP) on clinical documents for SARS-CoV-2 tests conducted outside of the VA. The algorithm to identify COVID-19 patients is continually updated to ensure new annotations of COVID-19 are captured from the clinical notes. For our analyses, we included those who have a record of being tested positive for SARS-CoV-2 in the VA healthcare system using the NST algorithm, which captured both asymptomatic and symptomatic patients.

We categorized participants by their COVID-19 disease state during the study period: hospitalization, ICU admission among those hospitalized, and death (among hospitalized and non-hospitalized). Individuals were included in all disease states they experienced, e.g. a patient who was hospitalized and then died afterwards would be categorized as both “hospitalized” as well as “died”. COVID-19-related hospitalizations were defined as hospital admissions between 7 days before and 30 days after an individual’s positive SARS-CoV-2 test. Mortality included all deaths up to 30 days after a positive test, with a maximum follow-up date of September 10, 2020. The index date for cases was defined as the date of first positive SARS-CoV-2 test and for non-cases was the date of first negative SARS-CoV-2 test, or August 10, 2020, which was the latest inclusion date for tested individuals, without a recorded test in the system by the NST algorithm.

### Comorbidities and phenotype description

Code-based phenotypes (PheCodes) were defined by manually grouping ICD-9 and ICD-10 diagnosis codes into clinically relevant groups by a clinical team for use in research as outlined in Denny et al. [32] PheCodes are mapped to a broader disease group which include circulatory, urinary, endocrine, symptoms, dermatologic, digestive, blood, sense, neurological, infectious, respiratory, and mental health diseases. A participant was considered to have the phenotype if they had ≥2 ICD-9 or ICD-10 codes for the phenotype in their medical record from up to 5 years prior to their index date. We only considered PheCodes Version 1.2b1 with prevalence of ≥5% in each comparison group, which resulted in 1,809 phenotypes used in our analyses.

We examined key complications among hospitalized patients with COVID-19 which included respiratory failure, myocardial infarction, stroke, pulmonary hypertension, embolism and/or thrombosis, and acute renal failure based on previous literature. Complications were defined as having at ≥1 diagnosis code within 30 days from the index date, and no code one year prior to ensure we were capturing incident complications. We also examined complications by race among SARS-CoV-2 positive individuals.

### Demographic and clinical characteristics

Demographic and clinical characteristics were obtained from the VA EHR housed within the VA’s Corporate Data Warehouse (CDW) [35] and the MVP central data repository, curated EHR and survey data available only for MVP research studies. Age, sex, race and ethnicity for participants were derived from the MVP Baseline Survey and supplemented with EHR data from CDW when self-reported demographics were not available [36]. Lifestyle factors including smoking history, alcohol consumption using the AUDIT-C screening test [37–40], homelessness and housing were extracted from the EHR, using VA registry and health factor data [41]. The health factors data contain responses to questionnaires administered during clinic visits that ask about a Veteran’s lifestyle behaviors. We considered a Veteran as from a nursing home if there was any admission to or from a VA Community Living Center or nursing home in 2019, or if a long-term care center was indicated around the time of the SARS-CoV-2 test. We defined those with an income of <$12,490 as below the 2019 Federal Poverty Level using their most recently reported income. Prior medication use was evaluated using the outpatient pharmacy indicated in the EHR up to one year prior to each participant’s index date. Blood pressure and heart rate measurements from the EHR between January 1, 2019 to December 31, 2019 were used, and the mean value using all measurements was reported. Body mass index was calculated using the average height and weight between January 1, 2017 to December 31, 2019.

### Statistical analysis

We examined baseline characteristics among the overall study sample, SARS-CoV-2 infected individuals and the stages of COVID-19 disease progression: hospitalization, ICU admission, and death.

We used logistic regression models to evaluate the association between each phenotype and each COVID-19 disease state: SARS-CoV-2 infection (Model 1); Hospitalized after COVID-19 diagnosis (Model 2); ICU admission after COVID-19 diagnosis (Model 3); and 30-day mortality after COVID-19 diagnosis (Model 4). In model 1, individuals without a positive SARS-CoV-2 test were considered non-cases, which includes those who were not tested and those who tested negative. Models 2–4 were restricted to patients with at least one SARS-CoV-2 positive test. All models were adjusted for age at index date, sex, race, ethnicity, state infection rate from USAfacts.org [42] during the corresponding week at index, and two measures of health utilization: the log-transformed number of months with a VA healthcare visit and the log-transformed total number of ICD-9/10 codes from 5 years prior to index date. We performed two sensitivity analyses for Model 1 restricting to: a) symptomatic COVID-19 cases only and b) those who received a SARS-CoV-2 lab in the VA.

Diagnostic tests for SARs-CoV-2 were not allocated randomly, and it is possible that ascertainment bias may impact estimates for models assessing COVID-19 disease outcomes. To evaluate ascertainment bias, we plotted the odds ratios for SARS-CoV-2 infection (Model 1) against odds ratios for being tested for SARs-CoV-2 (Model 5).

To account for multiple testing, we used the Benjamini-Hochberg procedure to control the false discovery rate (FDR) at ≤0.1 [43]. The FDR significance levels for SARS-CoV-2 infection, hospitalization, ICU, and death were set at 0.0095, 0.0028. 0.0006, 0.0002, respectively.

## Results and discussion

Demographic and clinical characteristics for the study base cohort and by COVID-19 disease progression stages are summarized in Table 1. Among 648,202 individuals in the base population, 5,929 tested positive for SARS-CoV-2 of which 4,029 (68%) were tested at the VA and 3,255 (55%) had at least one symptom recorded. We observed increasing age and higher proportion of men, former smokers, nursing home admissions, anti-hypertensive medication use, statins, diabetic agents, and respiratory agents with COVID-19 disease progression. For comorbidities, we observed higher crude prevalence of hypertension, myocardial infarction, diabetes, chronic respiratory disease, dementia, stroke, and renal failure with disease progression. We also observed a decreasing crude proportion of Hispanic individuals and AUDIT-C defined high-risk drinkers with COVID-19 disease progression. Hypertension was the most prevalent comorbidity among hospitalized cases followed by diabetes, chronic respiratory disease, and renal failure.

**Table 1:** Demographic and clinical characteristics of Million Veteran Program participants tested for SARS CoV-2 between March 1, 2020 and August 10, 2020.

	Base Cohort	SARS CoV-2+	COVID-19, Hospitalized^b^	COVID-19, ICU^b^	COVID-19, Death^b^
**Demographics**^**a**^	N = 648,202	N = 5,929	N = 1,463	N = 579	N = 398
Age, years	64.3 (14.1)	62.0 (14.6)	67.6 (12.1)	68.6 (11.2)	74.8 (10.4)
Men, n (%)	575225 (90%)	5213 (89%)	1368 (94%)	551 (96%)	385 (97%)
Women, n (%)	64506 (10%)	643 (11%)	86 (6%)	25 (4.3%)	12 (3.0%)
Race, n (%)					
White	465565 (72%)	3236 (55%)	760 (52%)	288 (50%)	243 (61%)
Black or African-American	124034 (19%)	2099 (35%)	596 (41%)	255 (44%)	131 (33%)
Other	58603 (9%)	594 (10%)	107 (7%)	36 (6.2%)	24 (6.0%)
Hispanic, n (%)	44041 (6.8%)	741 (12.5%)	152 (10.4%)	56 (9.7%)	36 (9.0%)
High-risk drinker, n (%)	86707 (13.4%)	661 (11.2%)	111 (7.6%)	39 (6.7%)	25 (6.3%)
Former smoker, n (%)	309661 (48%)	2797 (47%)	785 (54%)	328 (57%)	249 (63%)
Current smoker, n (%)	154687 (24%)	1115 (19%)	289 (20%)	113 (20%)	72 (18%)
Never smoker, n (%)	183854 (28%)	2017 (34%)	389 (27%)	138 (24%)	77 (19%)
Homeless, n (%)	43088 (6.6%)	624 (10.5%)	200 (13.7%)	62 (10.7%)	26 (6.5%)
Nursing home, n (%)	7072 (1.1%)	342 (5.8%)	150 (10.3%)	65 (11.2%)	48 (12.1%)
Hospital days 2019	1.6 (11.8)	4.5 (23.3)	10.0 (34.7)	10.1 (34.1)	10.0 (40.3)
Outpatient visit days 2019	24.7 (24.4)	35.6 (33.6)	47.3 (43.3)	46.9 (37.8)	45.7 (37.3)
Below poverty level, n (%)	116491 (21%)	1122 (22%)	319 (25%)	124 (24%)	73 (20%)
Region, n (%)					
Continental, n (%)	89997 (15%)	1008 (19%)	252 (20%)	88 (18%)	55 (16%)
Midwest, n (%)	95540 (16%)	632 (12%)	167 (13%)	74 (15%)	44 (13%)
North Atlantic, n (%)	130500 (22%)	1262 (24%)	319 (25%)	119 (24%)	116 (34%)
Pacific, n (%)	144558 (24%)	1046 (20%)	235 (19%)	99 (20%)	64 (19%)
Southeast, n (%)	134635 (23%)	1377 (26%)	284 (23%)	113 (23%)	58 (17%)
Urban, n (%)	434570 (73%)	4511 (85%)	1110 (88%)	431 (87%)	290 (86%)
Rural, n (%)	160660 (27%)	814 (15%)	147 (12%)	62 (13%)	47 (14%)
Body mass index, kg/m^2^	30.5 (6.0)	31.6 (6.3)	31.6 (6.8)	32.1 (7.0)	30.6 (7.0)
Systolic blood pressure, mm Hg	132 (14)	132 (13)	134 (14)	134 (13)	133 (14)
Diastolic blood pressure, mm Hg	77 (8)	77 (8)	76 (8)	76 (8)	73 (7)
Resting heart rate, beats/minute	75 (11)	76 (11)	77 (11)	77 (11)	75 (10)
**Comorbidities**					
Hypertension, n (%)	414658 (64%)	4091 (69%)	1198 (82%)	501 (87%)	349 (88%)
Myocardial infarction, n (%)	21568 (3.3%)	309 (5.2%)	133 (9.1%)	60 (10.4%)	42 (10.6%)
Diabetes mellitus, n (%)	248550 (38%)	2810 (47%)	868 (59%)	358 (62%)	239 (60%)
Chronic respiratory disease, n (%)	131983 (20%)	1412 (24%)	465 (32%)	200 (35%)	125 (31%)
Dementia, n (%)	16034 (2.5%)	364 (6.1%)	150 (10.3%)	46 (7.9%)	79 (19.8%)
Stroke, n (%)	31568 (4.9%)	420 (7.1%)	179 (12.2%)	80 (13.8%)	69 (17.3%)
Embolism, n (%)	1686 (0.3%)	19 (0.3%)	5 (0.3%)	1 (0.2%)	2 (0.5%)
Pulmonary hypertension, n (%)	1095 (0.2%)	19 (0.3%)	6 (0.4%)	5 (0.9%)	4 (1%)
Renal failure, n (%)	83953 (13%)	1178 (20%)	501 (34%)	224 (39%)	172 (43%)
Respiratory failure, n (%)	14627 (2.3%)	262 (4.4%)	141 (9.6%)	78 (13.5%)	53 (13.3%)
**Medications**					
Beta blocker, n (%)	177725 (27%)	1908 (32%)	619 (42%)	265 (46%)	183 (46%)
Alpha blocker, n (%)	157037 (24%)	1671 (28%)	506 (35%)	213 (37%)	154 (39%)
Calcium channel blocker, n (%)	144369 (22%)	1679 (28%)	513 (35%)	205 (35%)	138 (35%)
Insulin & diabetic agents, n (%)	160868 (25%)	1979 (33%)	621 (42%)	259 (45%)	161 (41%)
Statin, n (%)	320697 (50%)	3242 (55%)	945 (65%)	389 (67%)	251 (63%)
Respiratory agents, n (%)	141606 (22%)	1628 (28%)	495 (34%)	210 (36%)	125 (31%)

^a^All values are presented as mean (SD) unless otherwise specified.

^b^Participants can be included in more than one COVID-19 outcome of hospitalized, intensive care unit or death.

We observed a monotonic increase in risk for all COVID-19 outcomes with older age. Patients hospitalized for SARS-CoV-2 were more likely to be male, Black or African American, or obese (BMI ≥35 kg/m^2^) (S1 Fig).

Among the 5,929 individuals with SARS-CoV-2, 1,463 were hospitalized, of which 52% were White, 41% were Black and 7% were other races. Black hospitalized COVID-19 individuals had higher incidences of complications including respiratory failure (51% vs 42%), myocardial infarction (7.0% vs 6.1%), and acute renal failure (29% vs 18%) following a COVID-19 diagnosis compared to White individuals. White individuals experienced slightly higher 30-day mortality compared to Black individuals (7.5% vs. 6.2%). Black individuals were more likely to be admitted to the ICU (43% vs 38%), intubated (17% vs 12%), or readmitted to the hospital (7.2% vs. 6.6%) compared to White individuals (Table 2).

**Table 2:** Complications and adverse outcomes among SARS-CoV-2 positive individuals, stratified by race.

	White	Black or African American	Other
	N = 3236	N = 2099	N = 594
**Outcomes**			
Hospitalizations	760 (24%)	596 (28%)	107 (18%)
30-day Mortality (hospitalized and not hospitalized)	243 (7.5%)	131 (6.2%)	24 (4.0%)
**Complications among hospitalized patients**			
Respiratory failure	321 (42%)	306 (51%)	46 (43%)
Myocardial Infarction	46 (6.1%)	42 (7.0%)	6 (5.6%)
Stroke	25 (3.3%)	16 (2.7%)	1 (0.9%)
Pulmonary Hypertension	2 (0.3%)	1 (0.2%)	0 (0.0%)
Embolism and Thrombosis	3 (0.4%)	2 (0.3%)	0 (0.0%)
Acute Renal Failure	139 (18%)	172 (29%)	17 (16%)
No complications	398 (52%)	367 (62%)	51 (48%)
≥1 complication	362 (48%)	229 (38%)	56 (52%)
≥2 complications	116 (15%)	151 (25%)	17 (16%)
**Outcomes among hospitalized patients**			
Intensive Care Unit (ICU) admissions	288 (38%)	255 (43%)	36 (34%)
Intubation	93 (12%)	99 (17%)	15 (14%)
30-day discharge	553 (73%)	445 (75%)	82 (77%)
30-day readmission	50 (6.6%)	43 (7.2%)	7 (6.5%)
30-day mortality	145 (19%)	92 (15%)	13 (12%)

### Phenome-wide associations for COVID-19 disease progression

**Fig 2:**
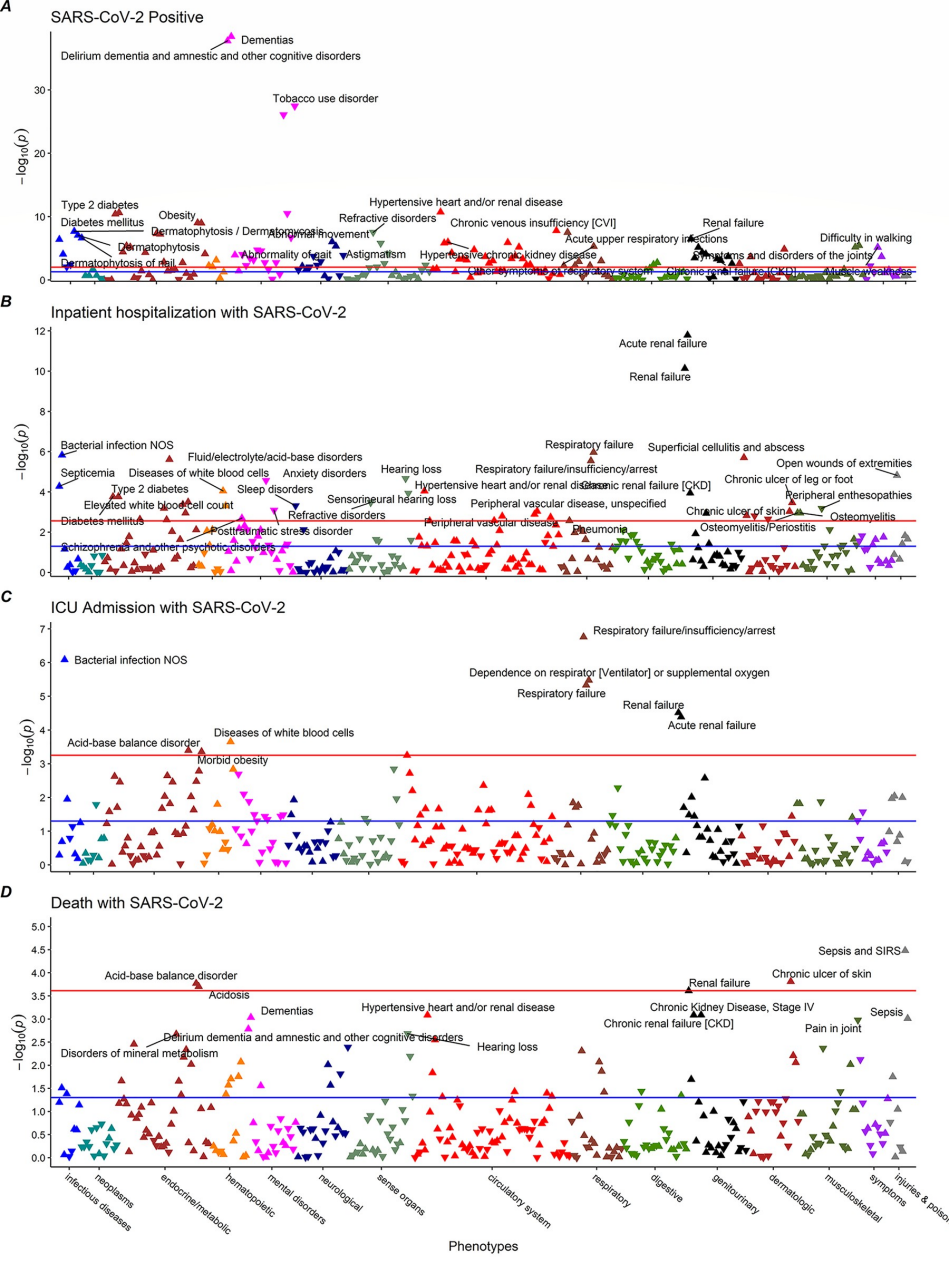
Phenome-wide associations with COVID-19 progression for (a) tested positive, (b) hospitalization, (c) intensive care unit admission, and (d) death.

Among the 5,929 SARS-CoV-2 positive individuals, 1,463 (24.6%) were hospitalized, 579 (9.8%) were admitted to the ICU, and 398 (6.7%) died where outcomes are not mutually exclusive per individual. Out of 1,809 phenotypes used in our analyses, 191, 48, 10, and 4 phenotypes were significantly associated with SARS-CoV-2, hospitalization, ICU, and death, respectively (Fig 2). The full list of significant phenotypes and corresponding disease groups can be found in S1 Table.

Among the significant associations, 26 phenotypes that were associated with at least two outcomes. A set of 6 phenotypes were associated with three outcomes: acute and non-acute renal failure, respiratory failure, chronic skin ulcers, acid-base balance disorder, and bacterial infections.

**Fig 3:**
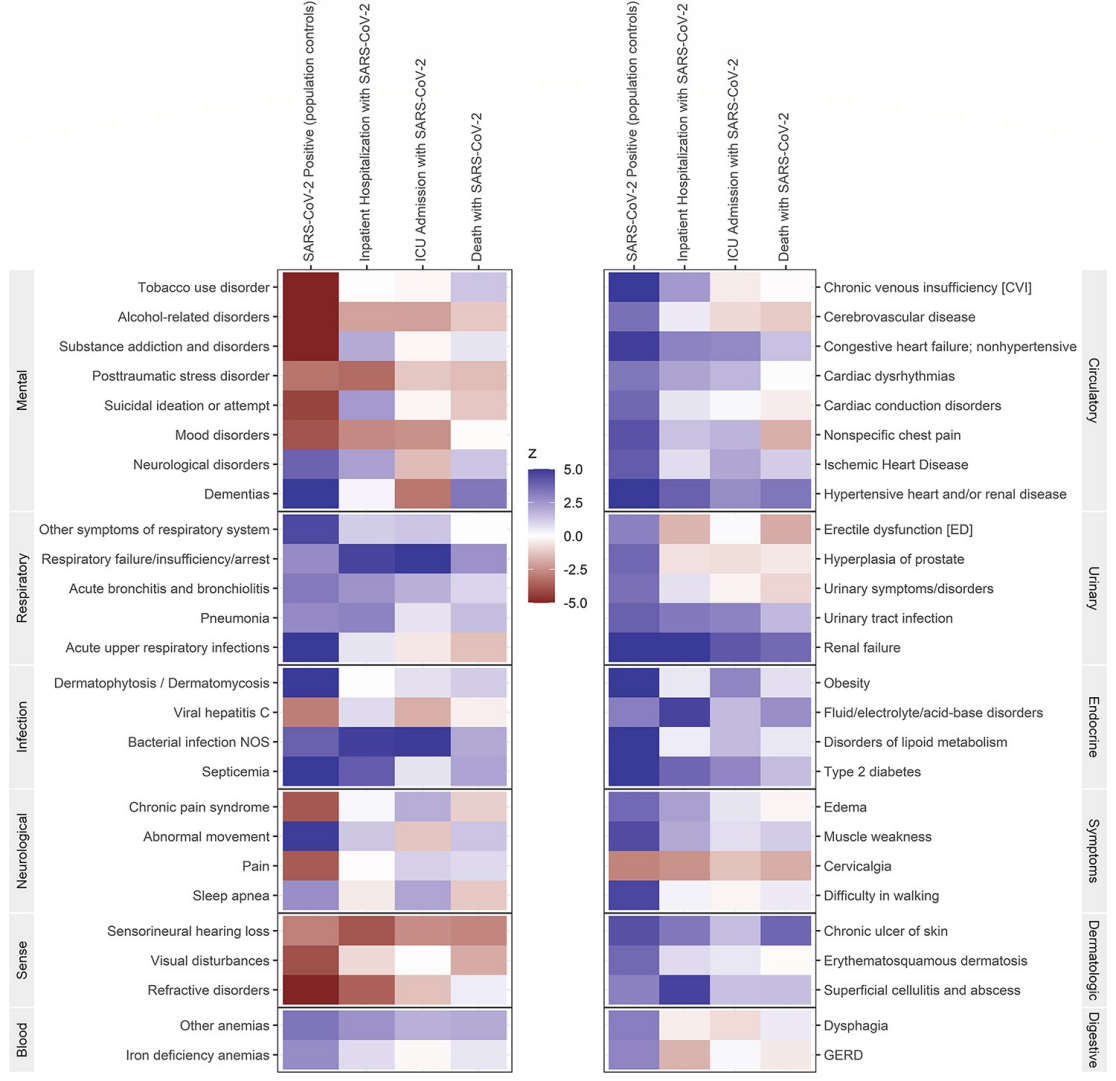
Heat map of phenotypes associated with COVID-19 outcomes. Blue indicates an increased risk and red indicates a decreased risk of the outcome.

Overall, we observed an increased risk of COVID-19 and its disease progression with a history of Circulatory, Endocrine, Respiratory, Urinary, and Dermatologic disease groups. The Heat Map (Fig 3) summarizes the associations between specific phenotypes with the four COVID-19 outcomes used in our analysis. The directionality (blue color for increased risk) of the phenotypic associations were mostly consistent across COVID-19 outcomes, with some attenuation for ICU and death due to low number of events. We observed that patients with prevalent congestive heart failure, ischemic heart disease, hypertensive heart and/or renal disease, obesity, fluid/electrolyte/acid-base disorders, disorder of lipoid metabolism, type 2 diabetes, respiratory failure/insufficiency/arrest, active bronchitis and bronchiolitis, pneumonia, urinary tract infection, renal failure, chronic ulcer of skin, and superficial cellulitis and abscess were associated with an increased risk across all COVID-19 disease stages. Mental health and sense disease groups overall were associated with a decreased risk of COVID-19 (red color for decreased risk) and the subsequent disease stages. Specifically, alcohol-related disorders, post-traumatic stress disorder, mood disorders, sensorineural hearing loss, visual disturbance, and refractive disorder with a few exceptions of substance addiction and disorders, neurological disorders, and dementia.

**Fig 4:**
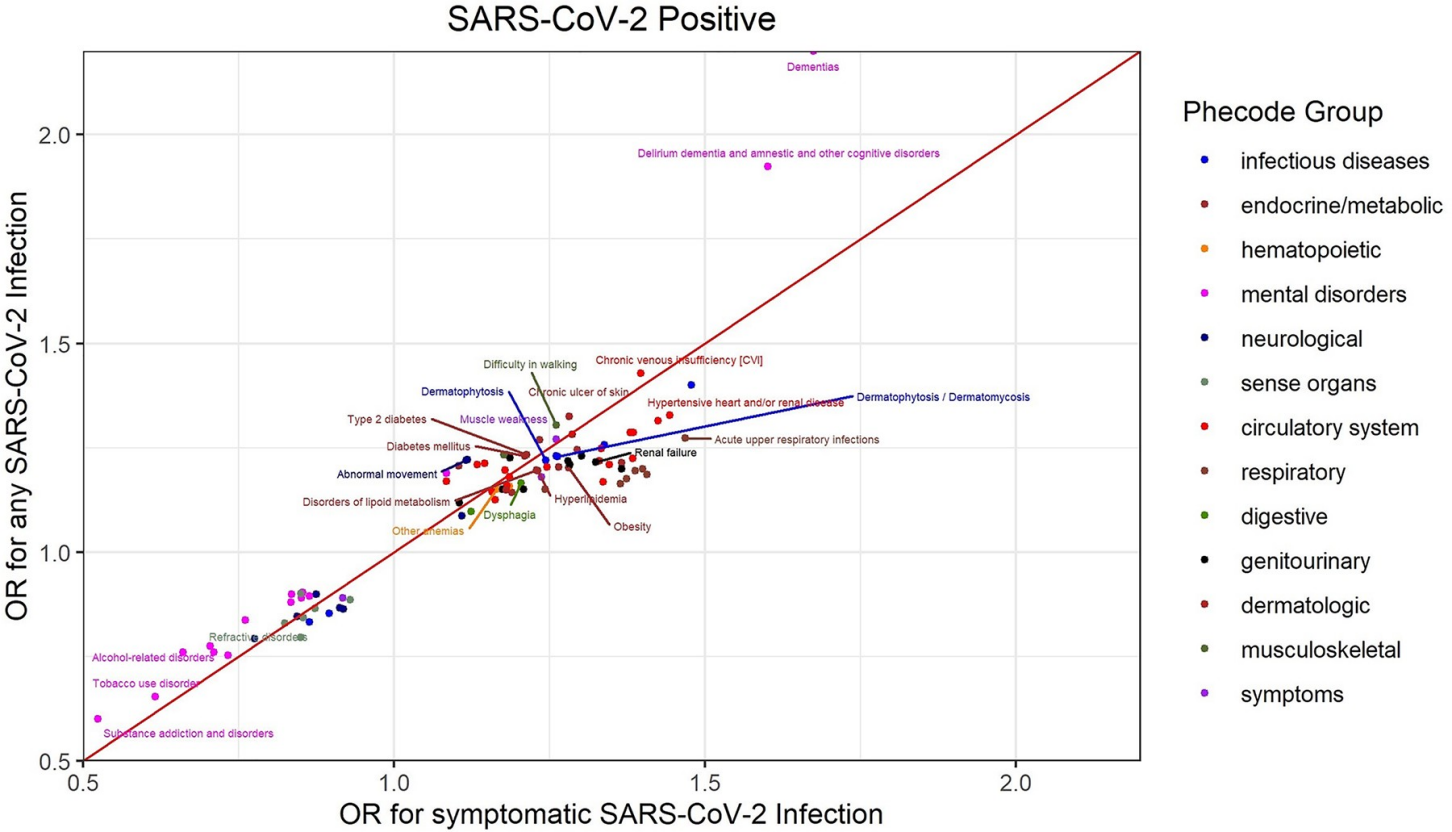
Comparison of odds ratio for symptomatic SARS-CoV-2 infection and odds ratio for asymptomatic or symptomatic SARS-CoV-2 infected individuals.

When we restricted our analyses to symptomatic COVID-19 cases, results were similar to Model 1 where all COVID-19 cases were included (Fig 4). A notable exception is dementia which had a higher odds ratio for SARS-CoV-2 when including asymptomatic and symptomatic cases compared to symptomatic cases only. We also observed similar results when restricting our analyses to COVID-19 cases diagnosed in the VA (data not shown).

### Ascertainment bias

The odds ratios of diseases from Model 5 (tested for SARS-CoV-2) and Model 1 (SARS-CoV-2 infection) were plotted together at two time points to assess if ascertainment bias may have impacted our observed results, and if it changed over time (S3 Fig). Most diseases that were positively associated with SARS-CoV-2 infection were also positively associated with receiving a SARS-CoV-2 test. Phenotypes close to the diagonal line had similar strength and direction of the effect size between infection and testing, which have the potential for ascertainment bias in studies restricted to COVID-19 patients or tested individuals. The diseases with low risk for ascertainment bias are those near the odds ratio for testing = 1.0 (not associated with receiving a test), such as substance addiction and disorders, Type 2 diabetes mellitus, obesity. In general, comorbidities were more strongly associated with testing earlier in the pandemic (June), but by August the testing was more uniform, i.e. most conditions moved closer to an OR = 1 for diagnostic testing. In particular, alcohol-related and tobacco-use disorders, dermatophytosis, and conditions that limit mobility were much less associated with testing by August.

Our study is the first longitudinal study to examine the phenome-wide associations of multiple comorbidities and critical stages of COVID-19 disease progression in a large cohort. Our results are consistent with previous studies that have shown that circulatory, endocrine, respiratory, and urinary disease groups are associated with a higher risk of COVID-19 [11, 44]. We also observed differences in characteristics and outcomes after COVID-19 infection by race that were consistent with previous reports [3, 4, 45]. Black individuals with COVID-19 had a greater incidence of renal failure, respiratory failure, multiple complications, ICU admissions and re-admission, intubation, and inpatient deaths following their COVID-19 diagnosis compared to White and Other race individuals. However, we observed that Hispanic individuals were more likely to be infected by less likely to have severe outcomes which may be due to incomplete information or small numbers [46].

The analysis revealed that individuals with dementia, other cognitive disorders, or conditions that may limit physical mobility had a higher risk of having COVID-19. Patients with these conditions may have difficulty maintaining social distancing as they require additional care from family members or clinical staff, which increases their potential exposure [47–49]. In our sensitivity analysis restricting to symptomatic COVID-19 patients only, those with dementia had the most notable difference in odds ratio compared to the model including asymptomatic and symptomatic COVID-19. The change in effect estimates may be a result of more frequent testing, regardless of symptoms, of dementia patients who may be more likely to be in nursing homes or underreporting of symptoms from the patient. We also observed that mental health conditions, including posttraumatic stress disorder, alcohol, tobacco and substance-use disorder, and sense diseases were associated with a lower risk of COVID-19. It is possible that those with these conditions were less likely to initiate tests for SARS-CoV-2, had difficulty reporting symptoms thus not captured as an infected person, or are more likely to self-isolate and thereby minimizing potential exposure [50]. However, due to the nature of our study design we cannot infer causality and only speculate the nature of the observed association.

In our assessment of ascertainment bias of COVID-19 cases, multiple major comorbidities had similar odds ratios for testing for SARS-CoV-2 and having SARS-CoV-2 infection, indicating SARS-CoV-2 testing was highly selective within the VA. During early months of the pandemic, testing was limited to patients showing COVID-19 related symptoms, or deemed at high-risk with a history of hypertension, diabetes or renal diseases. Downstream epidemiology and genetics studies should be aware that when selecting controls for observational analyses, patients who had a negative test for SARS-CoV-2 or visited the hospital during the COVID-19 pandemic likely have different clinical characteristics from the general population and could introduce sampling bias. Understanding such bias is important for accurately identifying causal risk factors, and underlying genetic determinants of disease incidence and progression, as in genome-wide association studies (GWAS). However, we observed that more diseases were not associated with getting tested in August compared to June suggesting that as testing became more widely available, the impact of ascertainment bias may be changing as the COVID-19 pandemic evolves overtime.

### Strengths and limitations

MVP has recruited 1 out of 8 Veteran users of the VHA network and current and previous studies that compared MVP to the general VA population has shown considerable agreement between the two groups [36]. Our analyses reproduced well known associations of comorbidities against COVID-19 and its complications. We also had a large multi-racial sample size that allowed us to examine the major COVID-19 disease stages with a broad set of phenotypes (>1800) and had a good distribution of cases in all U.S. regions. The VA EHR contains 20 years of clinical records, offering an extensive view of clinical characteristics, medications histories, vital signs and laboratory tests for COVID-19+ patients.

One limitation of the VA data is the lack of information on individuals tested outside the VA system. Such individuals may have been hospitalized elsewhere and records on SARS-CoV-2 infection or the exact date and timing of COVID-19 related outcomes may not be accurate as these data are based on administrative claims, and not actual dates of care. However, this lack of information would not affect the associations with COVID-19 or death. Similarly, not all patients visit the VA for every medical need, so disease domains defined using ICD-codes in the EHR may not fully capture an individual’s comorbidities. However, using a sample restricted to those who had a VA visit in 2019 and considering ICD codes from the previous 5 years may have reduced the impact of missing data from inactive VA users with incomplete medical histories in their EHR. Furthermore, we obtained similar results when restricting to VA COVID-19 cases (68% of all cases) in our model assessing the COVID-19 outcome.

All analyses were performed using the same set of general adjustment variables for consistency and do not account for all potential confounders and thus unmeasured or residual confounding could explain the observed associations. However, the phenome-wide association analyses were designed to be exploratory and intended to generate hypotheses toward understanding the progression of COVID-19 illness.

## Conclusions

Our large-scale phenome-wide approach identifies clusters of diseases which may be indicative of underlying biological mechanisms of COVID-19 disease severity and provides further insights for future observational, genomic, and multi-omic studies. Furthermore, identification of risk factors for different clinical stages of COVID-19 will help to optimize clinical management where recently approved drugs are limited and to prioritize critically ill COVID-19 patients.

## Supporting information

S1 Table Adjusted p-values for phenotypes associated with SARS-CoV-2, hospitalization, intensive care unit admission, and death with COVID-19. (DOCX)

S1 Fig Forest plot of odds ratios and 95% confidence intervals of key characteristics and COVID-19 disease states. (TIFF)

S2 Fig Association of 1809 phenotypes and testing for SARS-CoV-2. (TIFF)

S3 Fig COVID-19 ascertainment bias plot: Odds ratios of phenotypes for being tested for SARS-CoV-2 vs. being infected. (TIFF)

S1 File Million Veteran Program full acknowledgement. (DOCX)
