# Supplementary material for: Phenome-wide association of 1809 phenotypes and COVID-19 disease progression in the Veterans Health Administration Million Veteran Program
Source: PLoS One. 2021 May 13;16(5):e0251651. doi: 10.1371/journal.pone.0251651 (PMC8118298; doi:10.1371/journal.pone.0251651)
Supplement: S1 Table — (DOCX) [file pone.0251651.s001.docx]

**S1 Table.** **Adjusted p-values for phenotypes associated with SARS-CoV-2, hospitalization, intensive care unit admission, and death with COVID-19**

|  | **Adjusted P-value** | | | | | |
| --- | --- | --- | --- | --- | --- | --- |
| **Disease** | **Disease Group** | **SARS-CoV-2** | **Hospitalization** | **ICU** | **Death** |  |
| Sepsis and SIRS | injuries poisoning | **2.32E-04** | 1.48E-02 | 7.98E-01 | **3.30E-05** |  |
| Chronic ulcer of skin | dermatologic | **1.34E-05** | **9.26E-04** | 1.26E-01 | **1.54E-04** |  |
| Acid-base balance disorder | endocrine metabolic | 1.70E-01 | **4.18E-04** | **4.00E-04** | **1.69E-04** |  |
| Acidosis | endocrine metabolic | 2.90E-01 | **1.10E-03** | 9.40E-03 | **1.97E-04** |  |
| Renal failure | genitourinary | **3.34E-07** | **7.32E-11** | **3.04E-05** | 2.43E-04 |  |
| Chronic renal failure [CKD] | genitourinary | **6.92E-06** | **1.12E-04** | 1.98E-02 | 8.15E-04 |  |
| Hypertensive heart and/or renal disease | circulatory system | **1.89E-11** | **8.94E-05** | 6.27E-03 | 8.17E-04 |  |
| Dementias | mental disorders | **3.72E-39** | 8.08E-01 | 2.02E-03 | 9.20E-04 |  |
| Sepsis | injuries poisoning | **6.58E-05** | 2.18E-02 | 8.06E-01 | 9.72E-04 |  |
| Delirium dementia and amnestic and other co | mental disorders | **1.80E-38** | 9.03E-02 | 8.41E-02 | 1.64E-03 |  |
| Hearing loss | sense organs | 3.07E-02 | **2.15E-05** | 1.42E-03 | 2.07E-03 |  |
| Disorders of mineral metabolism | endocrine metabolic | 8.36E-01 | **6.47E-04** | 2.25E-03 | 2.13E-03 |  |
| Type 2 diabetes with renal manifestations | endocrine metabolic | **3.69E-05** | 6.75E-02 | 4.16E-01 | 3.48E-03 |  |
| Peripheral enthesopathies and allied syndrome | musculoskeletal | 6.55E-01 | **6.53E-04** | 1.34E-02 | 4.28E-03 |  |
| Decubitus ulcer | dermatologic | **4.22E-04** | 2.80E-01 | 5.62E-01 | 6.15E-03 |  |
| Hydrocephalus | neurological | **3.55E-04** | 8.59E-01 | 8.59E-01 | 6.22E-03 |  |
| Sensorineural hearing loss | sense organs | **4.40E-03** | **1.16E-04** | 1.09E-02 | 6.37E-03 |  |
| Disorders of fluid, electrolyte | endocrine metabolic | **1.79E-03** | **2.43E-06** | 1.09E-01 | 6.68E-03 |  |
| Respiratory failure, insufficiency, arrest | respiratory | **5.21E-03** | **2.82E-06** | **1.73E-07** | 8.47E-03 |  |
| Chronic ulcer of leg or foot | dermatologic | **8.27E-05** | **3.39E-04** | 3.58E-02 | 8.70E-03 |  |
| Other conditions of brain | neurological | **1.79E-03** | 2.85E-02 | 3.17E-01 | 9.71E-03 |  |
| Alzheimer's disease | mental disorders | **6.81E-09** | 3.94E-01 | 9.08E-02 | 1.21E-02 |  |
| Respiratory failure | respiratory | 5.23E-02 | **1.08E-06** | **4.62E-06** | 1.34E-02 |  |
| Hypertensive chronic kidney disease | circulatory system | **1.05E-06** | **2.72E-03** | 6.75E-02 | 1.45E-02 |  |
| Asphyxia and hypoxemia |  | **7.59E-03** | 5.34E-01 | 5.24E-02 | 1.57E-02 |  |
| Acute renal failure | genitourinary | **3.14E-04** | **1.63E-12** | **4.09E-05** | 2.02E-02 |  |
| Disorders of magnesium metabolism | endocrine metabolic | 8.04E-01 | **2.40E-03** | 1.51E-02 | 2.21E-02 |  |
| Eye infection, viral | sense organs | **3.60E-03** | 3.28E-01 | 2.31E-02 | 2.33E-02 |  |
| Varicose veins | circulatory system | **4.49E-04** | 9.50E-01 | 3.29E-02 | 2.41E-02 |  |
| Encephalopathy, not elsewhere classified | neurological | **7.90E-05** | 2.75E-02 | 3.75E-01 | 2.69E-02 |  |
| Altered mental status | mental disorders | **5.44E-06** | 6.69E-03 | 2.33E-01 | 2.78E-02 |  |
| Disturbance of skin sensation | dermatologic | 3.70E-01 | **1.55E-03** | 1.36E-02 | 2.78E-02 |  |
| Hydronephrosis | genitourinary | 1.21E-01 | 9.57E-03 | **4.30E-04** | 3.06E-02 |  |
| Septicemia | infectious diseases | **3.92E-07** | **5.21E-05** | 5.09E-01 | 3.08E-02 |  |
| Heart failure with reduced EF [Systolic or | circulatory system | **1.58E-04** | 8.17E-03 | 2.41E-02 | 3.73E-02 |  |
| Dependence on respirator [Ventilator] or su | respiratory | **1.89E-03** | 3.26E-02 | **3.33E-06** | 3.79E-02 |  |
| Bacterial infection NOS | infectious diseases | **8.41E-05** | **1.47E-06** | **8.19E-07** | 4.14E-02 |  |
| Other anemias | hematopoietic | **7.20E-04** | 8.53E-03 | 6.22E-02 | 4.23E-02 |  |
| Peripheral vascular disease, unspecified | circulatory system | 2.94E-02 | **9.06E-04** | 1.71E-02 | 4.66E-02 |  |
| Schizophrenia and other psychotic disorders | mental disorders | **8.63E-03** | **2.00E-03** | 9.61E-01 | 5.05E-02 |  |
| Secondary diabetes mellitus | endocrine metabolic | **1.78E-04** | 6.87E-01 | 9.43E-01 | 5.36E-02 |  |
| Congestive heart failure (CHF) NOS | circulatory system | **4.29E-04** | 4.26E-02 | 6.00E-02 | 5.64E-02 |  |
| Visual disturbances | sense organs | **6.49E-05** | 4.09E-01 | 9.44E-01 | 5.94E-02 |  |
| Varicose veins of lower extremity | circulatory system | **1.26E-03** | 7.18E-01 | 6.92E-02 | 6.06E-02 |  |
| Other hypertrophic and atrophic conditions | dermatologic | 6.97E-01 | **2.21E-03** | 6.93E-02 | 6.12E-02 |  |
| Erectile dysfunction [ED] | genitourinary | **2.50E-03** | 9.92E-02 | 8.97E-01 | 6.14E-02 |  |
| Chronic Kidney Disease, Stage III | genitourinary | **4.21E-05** | 1.18E-02 | 3.28E-02 | 6.23E-02 |  |
| Cervicalgia | symptoms | **5.81E-03** | 1.50E-02 | 1.73E-01 | 6.54E-02 |  |
| Diabetes mellitus | endocrine metabolic | **3.82E-11** | **1.72E-04** | 2.35E-03 | 6.82E-02 |  |
| Dermatophytosis of nail | infectious diseases | **2.32E-07** | 2.15E-01 | 5.55E-02 | 7.22E-02 |  |
| Nonspecific chest pain | circulatory system | **1.66E-05** | 1.45E-01 | 7.38E-02 | 7.53E-02 |  |
| Human immunodeficiency virus [HIV] disease | infectious diseases | **2.82E-06** | 8.42E-01 | 1.87E-01 | 7.96E-02 |  |
| Diabetes type 2 with peripheral circulatory | endocrine metabolic | **6.31E-08** | 1.64E-02 | 5.29E-01 | 8.07E-02 |  |
| Morbid obesity | endocrine metabolic | **7.25E-05** | 7.53E-03 | **4.36E-04** | 8.19E-02 |  |
| HIV infection, symptomatic | infectious diseases | **3.77E-06** | 7.72E-01 | 1.99E-01 | 8.22E-02 |  |
| Pruritus and related conditions | dermatologic | **8.88E-03** | 5.35E-01 | 2.87E-01 | 8.44E-02 |  |
| Peripheral vascular disease | circulatory system | 7.30E-02 | **1.27E-03** | 8.15E-03 | 8.53E-02 |  |
| Dementia with cerebral degenerations | mental disorders | **4.74E-10** | 5.07E-01 | 3.83E-01 | 8.55E-02 |  |
| Hypovolemia | endocrine metabolic | 1.21E-01 | **3.17E-04** | 2.32E-02 | 8.69E-02 |  |
| Open wounds of extremities | injuries poisoning | 2.04E-01 | **1.49E-05** | 2.02E-01 | 8.91E-02 |  |
| Urinary tract infection | genitourinary | **8.18E-05** | **1.14E-03** | 2.65E-03 | 9.85E-02 |  |
| Hypertensive heart disease | circulatory system | **1.41E-06** | 4.82E-02 | 1.80E-01 | 1.03E-01 |  |
| Sexually transmitted infections (not HIV or | infectious diseases | **1.30E-05** | 1.46E-01 | 2.12E-02 | 1.11E-01 |  |
| Osteomyelitis | musculoskeletal | 7.97E-01 | **1.10E-03** | 1.51E-02 | 1.13E-01 |  |
| Osteomyelitis, periostitis, and other infection | musculoskeletal | 8.41E-01 | **1.10E-03** | 1.51E-02 | 1.13E-01 |  |
| Parkinson's disease | neurological | **8.37E-04** | 6.30E-01 | 5.85E-01 | 1.15E-01 |  |
| Type 2 diabetes with neurological manifestation | endocrine metabolic | **6.47E-06** | 3.38E-02 | 1.59E-01 | 1.16E-01 |  |
| Type 2 diabetes | endocrine metabolic | **2.29E-11** | **1.71E-04** | 3.45E-03 | 1.19E-01 |  |
| Other specified peripheral vascular disease | circulatory system | 9.61E-01 | **2.42E-03** | 3.43E-01 | 1.20E-01 |  |
| Staphylococcus infections | infectious diseases | **7.44E-03** | 6.86E-02 | 1.11E-02 | 1.21E-01 |  |
| Chronic pain | neurological | **1.19E-03** | 8.78E-01 | 4.15E-01 | 1.21E-01 |  |
| Chronic kidney disease, Stage I or II | genitourinary | **2.55E-03** | 6.55E-02 | 3.59E-02 | 1.24E-01 |  |
| Pneumonia | respiratory | **4.90E-03** | **2.66E-03** | 5.04E-01 | 1.26E-01 |  |
| Varicose veins of lower extremity, symptomt | circulatory system | **9.08E-04** | 9.38E-01 | 1.51E-01 | 1.29E-01 |  |
| Chondromalacia | musculoskeletal | **1.98E-04** | 1.30E-01 | 3.94E-01 | 1.30E-01 |  |
| Superficial cellulitis and abscess | dermatologic | **2.54E-03** | **1.95E-06** | 1.37E-01 | 1.30E-01 |  |
| screening for infectious and parasitic disease |  | **5.63E-04** | 7.44E-01 | 2.44E-01 | 1.36E-01 |  |
| Type 2 diabetes with ophthalmic manifestation | endocrine metabolic | **4.04E-06** | 4.48E-01 | 2.72E-01 | 1.41E-01 |  |
| Posttraumatic stress disorder | mental disorders | **1.75E-03** | **8.10E-04** | 2.12E-01 | 1.43E-01 |  |
| Congestive heart failure; nonh | circulatory system | **1.21E-06** | **2.66E-03** | 4.36E-03 | 1.43E-01 |  |
| Other cerebral degenerations | neurological | **1.39E-07** | 5.65E-01 | 1.83E-01 | 1.58E-01 |  |
| Dysthymic disorder | mental disorders | **1.72E-04** | 6.06E-01 | 2.56E-01 | 1.59E-01 |  |
| Hypermetropia | sense organs | **2.50E-03** | 2.74E-02 | 5.11E-02 | 1.59E-01 |  |
| Cerebral degeneration, unspecified | neurological | **2.63E-06** | 6.37E-01 | 4.24E-01 | 1.60E-01 |  |
| Acute upper respiratory infections of multi | respiratory | **3.02E-08** | 5.40E-01 | 6.03E-01 | 1.61E-01 |  |
| Late effects of cerebrovascular disease | circulatory system | **7.52E-07** | 9.58E-01 | 4.14E-01 | 1.62E-01 |  |
| Suicidal ideation | mental disorders | **2.55E-05** | 7.71E-03 | 4.15E-01 | 1.63E-01 |  |
| Abnormal movement | neurological | **9.06E-07** | 1.90E-01 | 2.01E-01 | 1.68E-01 |  |
| Tobacco use disorder | mental disorders | **3.50E-28** | 9.61E-01 | 8.63E-01 | 1.72E-01 |  |
| Retention of urine | genitourinary | **6.36E-03** | 9.50E-02 | 2.13E-01 | 1.75E-01 |  |
| Neurological disorders | mental disorders | **1.12E-04** | 2.54E-02 | 1.38E-01 | 1.75E-01 |  |
| Corneal opacity and other disorders of corn | sense organs | **3.71E-03** | 8.76E-01 | 1.67E-01 | 1.76E-01 |  |
| Heart failure with preserved EF [Diastolic | circulatory system | **6.42E-06** | **1.60E-03** | 2.31E-02 | 1.78E-01 |  |
| Chronic dermatitis due to solar radiation | dermatologic | **2.38E-04** | 3.08E-02 | 1.37E-01 | 1.79E-01 |  |
| Suicidal ideation or attempt | mental disorders | **1.79E-05** | 1.57E-02 | 8.34E-01 | 2.09E-01 |  |
| Abnormality of gait | neurological | **4.38E-06** | 8.34E-02 | 2.18E-01 | 2.23E-01 |  |
| Other chronic ischemic heart disease, unspecified | circulatory system | **6.69E-04** | 8.05E-01 | 9.19E-01 | 2.25E-01 |  |
| Sleep apnea | neurological | **6.56E-03** | 6.42E-01 | 3.24E-02 | 2.30E-01 |  |
| Muscle weakness | symptoms | **6.79E-06** | 4.17E-02 | 4.37E-01 | 2.30E-01 |  |
| Keratoderma, acquired | dermatologic | **1.17E-03** | 1.53E-01 | 6.43E-01 | 2.31E-01 |  |
| Alcohol-related disorders | mental disorders | **3.15E-11** | 3.87E-02 | 3.58E-02 | 2.32E-01 |  |
| Urinary incontinence | genitourinary | **8.31E-04** | 6.67E-01 | 5.85E-01 | 2.33E-01 |  |
| Occlusion of cerebral arteries | circulatory system | **3.78E-03** | 2.35E-01 | 2.45E-01 | 2.34E-01 |  |
| Ischemic Heart Disease | circulatory system | **5.22E-05** | 4.30E-01 | 3.31E-02 | 2.39E-01 |  |
| Acute sinusitis | respiratory | **5.67E-03** | 5.16E-01 | 5.74E-01 | 2.40E-01 |  |
| Dermatophytosis / Dermatomycos | infectious diseases | **2.06E-08** | 9.69E-01 | 4.73E-01 | 2.40E-01 |  |
| Cerebral artery occlusion, with cerebral in | circulatory system | **3.48E-03** | 2.06E-01 | 2.28E-01 | 2.42E-01 |  |
| Cerebrovascular disease | circulatory system | **4.46E-04** | 6.28E-01 | 4.09E-01 | 2.46E-01 |  |
| Dermatophytosis | infectious diseases | **8.59E-08** | 8.21E-01 | 6.48E-01 | 2.50E-01 |  |
| Psychosis | mental disorders | **7.66E-03** | 9.46E-02 | 7.77E-01 | 2.51E-01 |  |
| Open wound of foot except toe(s) alone | injuries poisoning | 5.26E-02 | **1.61E-03** | 2.44E-01 | 2.64E-01 |  |
| Cellulitis and abscess of leg, except foot | dermatologic | **2.85E-04** | **1.48E-03** | 8.38E-01 | 2.65E-01 |  |
| Methicillin resistant Staphylococcus aureus | infectious diseases | **1.17E-03** | 2.36E-01 | 1.60E-01 | 2.80E-01 |  |
| Chronic pain syndrome | neurological | **1.30E-04** | 8.48E-01 | 5.31E-02 | 2.86E-01 |  |
| Pain in limb |  | **4.96E-03** | 7.83E-01 | 7.28E-01 | 2.88E-01 |  |
| Persons with potential health hazards relation |  | **2.94E-13** | 6.54E-01 | 4.82E-01 | 2.97E-01 |  |
| Dermatomycoses | infectious diseases | **1.68E-03** | 5.01E-01 | 8.28E-02 | 3.00E-01 |  |
| Diabetic retinopathy | endocrine metabolic | **4.54E-05** | 7.22E-01 | 7.02E-01 | 3.06E-01 |  |
| Personality disorders | mental disorders | **2.97E-04** | 2.24E-01 | 6.81E-01 | 3.14E-01 |  |
| Acute bronchitis and bronchiolitis | respiratory | **1.18E-03** | 1.04E-02 | 6.53E-02 | 3.31E-01 |  |
| Constipation |  | **2.73E-03** | 1.16E-01 | 4.21E-02 | 3.41E-01 |  |
| Alcoholism | mental disorders | **1.95E-07** | 4.58E-01 | 3.24E-02 | 3.49E-01 |  |
| Arrhythmia (cardiac) NOS | circulatory system | **4.08E-03** | 1.25E-01 | 2.33E-02 | 3.56E-01 |  |
| Other symptoms/disorders or the urinary sys | genitourinary | **4.71E-04** | 4.68E-01 | 7.95E-01 | 3.60E-01 |  |
| Osteoarthrosis, localized, secondary | musculoskeletal | 6.73E-01 | **1.94E-03** | 6.51E-01 | 3.68E-01 |  |
| Pain | neurological | **1.78E-04** | 9.46E-01 | 2.68E-01 | 3.82E-01 |  |
| Unstable angina (intermediate coronary syndrome | circulatory system | **1.42E-03** | 9.16E-01 | 2.63E-01 | 3.92E-01 |  |
| Infection of the eye | sense organs | **1.21E-03** | 1.89E-01 | 7.94E-01 | 4.21E-01 |  |
| Coronary atherosclerosis | circulatory system | **3.91E-04** | 7.04E-01 | 1.81E-01 | 4.53E-01 |  |
| Overweight, obesity and other hyperalimenta | endocrine metabolic | **9.56E-10** | 5.81E-01 | 3.32E-03 | 4.55E-01 |  |
| Agorophobia, social phobia, and panic disorder | mental disorders | **8.67E-03** | 1.60E-01 | 2.78E-02 | 4.65E-01 |  |
| Dermatophytosis of the body | infectious diseases | **3.21E-04** | 2.28E-01 | 1.61E-02 | 4.73E-01 |  |
| Other signs and symptoms involving emotional |  | **1.63E-03** | 3.03E-01 | 5.48E-01 | 4.74E-01 |  |
| Hypercholesterolemia | endocrine metabolic | **1.46E-03** | 7.73E-02 | 5.08E-01 | 4.86E-01 |  |
| Obesity | endocrine metabolic | **1.04E-09** | 4.58E-01 | 1.64E-03 | 4.98E-01 |  |
| Other abnormality of urination | genitourinary | **9.52E-03** | 6.32E-01 | 7.94E-01 | 5.09E-01 |  |
| Other tests |  | **4.92E-03** | 9.21E-02 | 5.27E-02 | 5.19E-01 |  |
| Lack of coordination | neurological | **6.10E-03** | 9.07E-01 | 2.70E-01 | 5.25E-01 |  |
| Vascular dementia | mental disorders | **1.39E-15** | 5.49E-01 | 4.52E-02 | 5.34E-01 |  |
| Symptoms involving nervous and musculoskele | symptoms | **6.39E-03** | 7.89E-01 | 8.33E-01 | 5.42E-01 |  |
| Substance addiction and disorders | mental disorders | **8.27E-27** | 4.75E-02 | 8.60E-01 | 5.44E-01 |  |
| Congenital anomalies of great vessels | congenital anomalie | **2.90E-03** | 9.23E-01 | 9.61E-01 | 5.50E-01 |  |
| Hyperlipidemia | endocrine metabolic | **5.73E-08** | 6.65E-01 | 1.03E-01 | 5.59E-01 |  |
| Other specified diseases of sebaceous gland | dermatologic | **2.40E-04** | 4.37E-01 | 1.12E-01 | 5.60E-01 |  |
| Iron deficiency anemias | hematopoietic | **5.36E-03** | 4.11E-01 | 8.76E-01 | 5.63E-01 |  |
| Superficial injury without mention of infec |  | **5.88E-03** | 2.70E-01 | 7.45E-01 | 5.67E-01 |  |
| Anxiety disorders | mental disorders | **1.77E-03** | **2.70E-05** | 3.63E-02 | 5.67E-01 |  |
| Contusion | injuries poisoning | **1.86E-03** | 2.19E-01 | 1.31E-01 | 5.82E-01 |  |
| Disorders of lipoid metabolism | endocrine metabolic | **4.77E-08** | 6.58E-01 | 1.11E-01 | 5.83E-01 |  |
| Suicide or self-inflicted injury | mental disorders | **2.16E-03** | 1.02E-01 | 9.50E-01 | 5.84E-01 |  |
| Hyperplasia of prostate | genitourinary | **1.91E-04** | 5.10E-01 | 4.63E-01 | 6.00E-01 |  |
| GERD | digestive | **3.46E-03** | 9.12E-02 | 8.58E-01 | 6.28E-01 |  |
| Difficulty in walking | musculoskeletal | **3.46E-06** | 7.66E-01 | 8.06E-01 | 6.29E-01 |  |
| Dysphagia | digestive | **1.78E-03** | 7.05E-01 | 4.27E-01 | 6.29E-01 |  |
| Encounter for long-term (curre | circulatory system | **1.73E-03** | 6.62E-01 | 4.94E-01 | 6.29E-01 |  |
| Other diseases of respiratory system, not e | respiratory | **2.86E-03** | 5.83E-02 | 3.55E-01 | 6.30E-01 |  |
| Symptoms and disorders of the joints | musculoskeletal | **6.36E-06** | 8.23E-01 | 5.00E-01 | 6.36E-01 |  |
| Urethral discharge | genitourinary | **5.07E-03** | 8.95E-01 | 5.59E-01 | 6.37E-01 |  |
| Other heart block | circulatory system | **2.21E-05** | 5.93E-01 | 4.97E-01 | 6.50E-01 |  |
| Skull and face fracture and other intercran | injuries poisoning | **8.25E-03** | 2.97E-01 | 6.74E-01 | 6.64E-01 |  |
| Pyelonephritis | genitourinary | **5.28E-03** | 3.72E-02 | 2.32E-02 | 6.66E-01 |  |
| Cerebral atherosclerosis | circulatory system | **5.95E-03** | 9.67E-01 | 3.49E-01 | 6.72E-01 |  |
| Disorders of refraction and accommodation; | sense organs | **2.98E-08** | **3.35E-04** | 1.68E-01 | 6.79E-01 |  |
| Cardiac conduction disorders | circulatory system | **1.95E-04** | 5.40E-01 | 8.84E-01 | 6.80E-01 |  |
| Pneumonitis due to inhalation of food or vo | respiratory | **1.90E-03** | 3.62E-01 | 3.30E-01 | 6.82E-01 |  |
| Astigmatism | sense organs | **1.56E-06** | 2.29E-02 | 4.12E-02 | 6.94E-01 |  |
| Iron deficiency anemias, unspecified or not | hematopoietic | **8.12E-03** | 4.86E-01 | 4.98E-01 | 6.99E-01 |  |
| Viral hepatitis C | infectious diseases | **3.73E-03** | 4.19E-01 | 7.24E-02 | 7.17E-01 |  |
| Bipolar | mental disorders | **5.01E-03** | 2.07E-01 | 9.91E-02 | 7.38E-01 |  |
| Strabismus and other disorders of binocular | sense organs | **9.84E-04** | 4.14E-01 | 4.04E-01 | 7.40E-01 |  |
| Symptoms concerning nutrition, metabolism, |  | **4.86E-04** | 7.13E-01 | 4.41E-02 | 7.46E-01 |  |
| Hypotension | circulatory system | 1.33E-01 | **1.81E-03** | 2.13E-01 | 7.52E-01 |  |
| Strabismus (not specified as paralytic) | sense organs | **7.03E-03** | 4.25E-01 | 8.88E-01 | 7.55E-01 |  |
| Bone cancer | neoplasms | **3.60E-04** | 9.62E-01 | 9.62E-01 | 7.83E-01 |  |
| Major depressive disorder | mental disorders | **6.63E-04** | 5.51E-02 | 4.95E-02 | 7.83E-01 |  |
| Candidiasis | infectious diseases | 8.61E-01 | **1.79E-03** | 7.76E-03 | 7.95E-01 |  |
| Dermatitis due to solar radiation | dermatologic | **1.42E-08** | 2.71E-01 | 7.89E-02 | 7.99E-01 |  |
| Myopia | sense organs | **8.72E-03** | 6.50E-01 | 5.23E-01 | 8.04E-01 |  |
| Septal Deviations/Turbinate Hypertrophy | respiratory | **6.80E-03** | 8.34E-01 | 6.25E-01 | 8.08E-01 |  |
| Presbyopia |  | **7.42E-08** | **4.69E-04** | 2.36E-02 | 8.09E-01 |  |
| Edema | symptoms | **2.00E-04** | 2.28E-02 | 5.30E-01 | 8.18E-01 |  |
| Other symptoms |  | **2.48E-04** | 7.54E-01 | 4.65E-01 | 8.46E-01 |  |
| Duodenitis | digestive | **3.40E-03** | 1.98E-01 | 8.99E-01 | 8.47E-01 |  |
| Calculus of ureter | genitourinary | **8.82E-03** | 1.37E-01 | 3.60E-01 | 8.61E-01 |  |
| Bacteremia | infectious diseases | **6.02E-03** | 3.29E-03 | 2.01E-01 | 8.62E-01 |  |
| Dental abrasion, erosion and attrition | digestive | **4.57E-04** | 5.67E-01 | 8.34E-01 | 8.70E-01 |  |
| Facial weakness | symptoms | **4.27E-03** | 9.76E-01 | 7.82E-01 | 8.78E-01 |  |
| Balanoposthitis | genitourinary | **1.82E-03** | 8.53E-01 | 9.33E-01 | 8.85E-01 |  |
| Elevated white blood cell count | hematopoietic | 1.58E-01 | **4.90E-04** | 1.44E-03 | 8.91E-01 |  |
| Acute pain | neurological | **4.60E-03** | 7.46E-01 | 2.74E-01 | 8.95E-01 |  |
| screening for malignant neoplasms |  | **4.46E-04** | 3.00E-01 | 7.85E-01 | 9.10E-01 |  |
| Basal cell carcinoma | neoplasms | **8.60E-03** | 9.44E-01 | 7.29E-01 | 9.14E-01 |  |
| Conjunctivitis, infectious | sense organs | **7.50E-03** | 3.60E-01 | 1.27E-01 | 9.20E-01 |  |
| Erythematosquamous dermatosis | dermatologic | **2.17E-04** | 3.94E-01 | 5.74E-01 | 9.23E-01 |  |
| Cardiac and circulatory congenital anomalies | congenital anomalies | **3.50E-03** | 7.57E-01 | 8.35E-01 | 9.27E-01 |  |
| Abnormal electrocardiogram [ECG] [EKG] | circulatory system | **2.21E-03** | 1.26E-01 | 2.72E-01 | 9.28E-01 |  |
| Myalgia and myositis unspecified | symptoms | **6.48E-03** | 9.99E-01 | 3.53E-01 | 9.28E-01 |  |
| Chronic venous insufficiency [CVI] | circulatory system | **1.61E-08** | 1.48E-02 | 6.60E-01 | 9.31E-01 |  |
| Mood disorders | mental disorders | **9.32E-05** | 7.67E-03 | 1.31E-02 | 9.39E-01 |  |
| Cardiac dysrhythmias | circulatory system | **8.95E-04** | 2.97E-02 | 8.60E-02 | 9.40E-01 |  |
| Viral hepatitis | infectious diseases | **8.87E-03** | 5.58E-01 | 1.61E-01 | 9.41E-01 |  |
| Sleep disorders | neurological | 2.68E-02 | **4.85E-04** | 2.58E-01 | 9.58E-01 |  |
| Cough | respiratory | **9.45E-04** | 4.71E-01 | 8.32E-01 | 9.58E-01 |  |
| Diseases of white blood cells | hematopoietic | 2.15E-01 | **8.81E-05** | **2.21E-04** | 9.64E-01 |  |
| Other symptoms of respiratory system | respiratory | **4.89E-06** | 2.38E-01 | 1.73E-01 | 9.64E-01 |  |
| Villonodular synovitis | musculoskeletal | **4.30E-03** | 6.12E-01 | 9.67E-01 | 9.69E-01 |  |
| Antirheumatics causing adverse | injuries poisoning | **1.29E-03** | 2.99E-01 | 3.82E-01 | 9.70E-01 |  |
| Angina pectoris | circulatory system | **5.09E-04** | 7.62E-01 | 2.02E-01 | 9.70E-01 |  |
| Depression | mental disorders | **3.66E-04** | 2.87E-02 | 3.20E-02 | 9.72E-01 |  |
| Insomnia | neurological | **8.52E-03** | 7.39E-03 | 1.25E-01 | 9.72E-01 |  |
| Acquired acanthosis nigricans | dermatologic | **6.06E-03** | 7.30E-01 | 9.62E-01 | 9.75E-01 |  |
| Congenital anomaly of fingers/toes | congenital anomalies | **2.74E-04** | 8.27E-01 | 9.65E-01 | 9.76E-01 |  |
| Seborrheic dermatitis | dermatologic | **2.55E-04** | 4.04E-01 | 5.18E-01 | 9.89E-01 |  |
| Other specified erythematous conditions | dermatologic | **2.95E-04** | 2.91E-01 | 9.28E-01 | 9.90E-01 |  |

*Bold p-values indicate statistically significant association
