# Supplementary figures and images for: Phenome-wide association of 1809 phenotypes and COVID-19 disease progression in the Veterans Health Administration Million Veteran Program
Source: PLoS One. 2021 May 13;16(5):e0251651. doi: 10.1371/journal.pone.0251651 (PMC8118298; doi:10.1371/journal.pone.0251651)

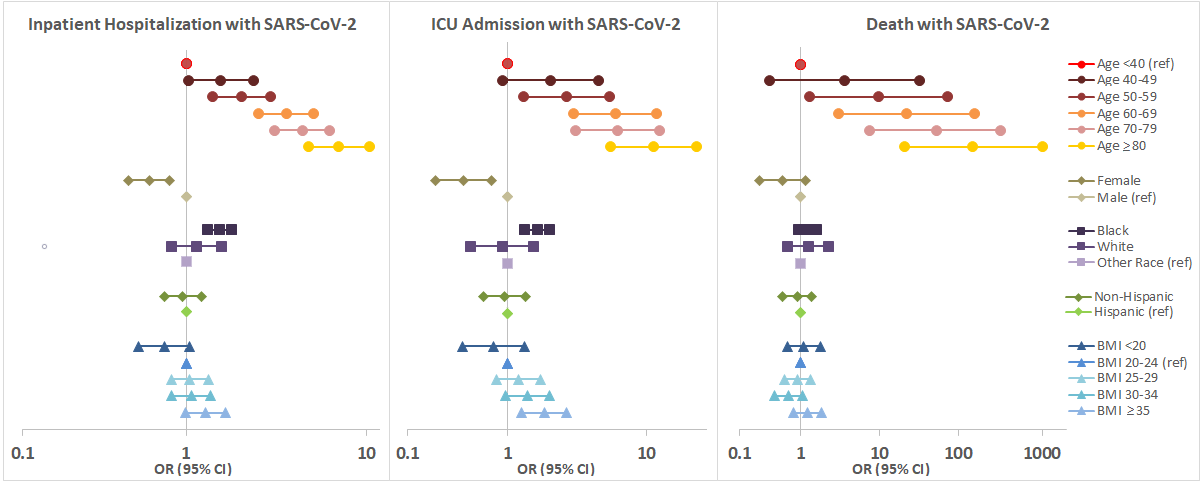

Supplement: S1 Fig — (TIFF) [file pone.0251651.s002.tiff]

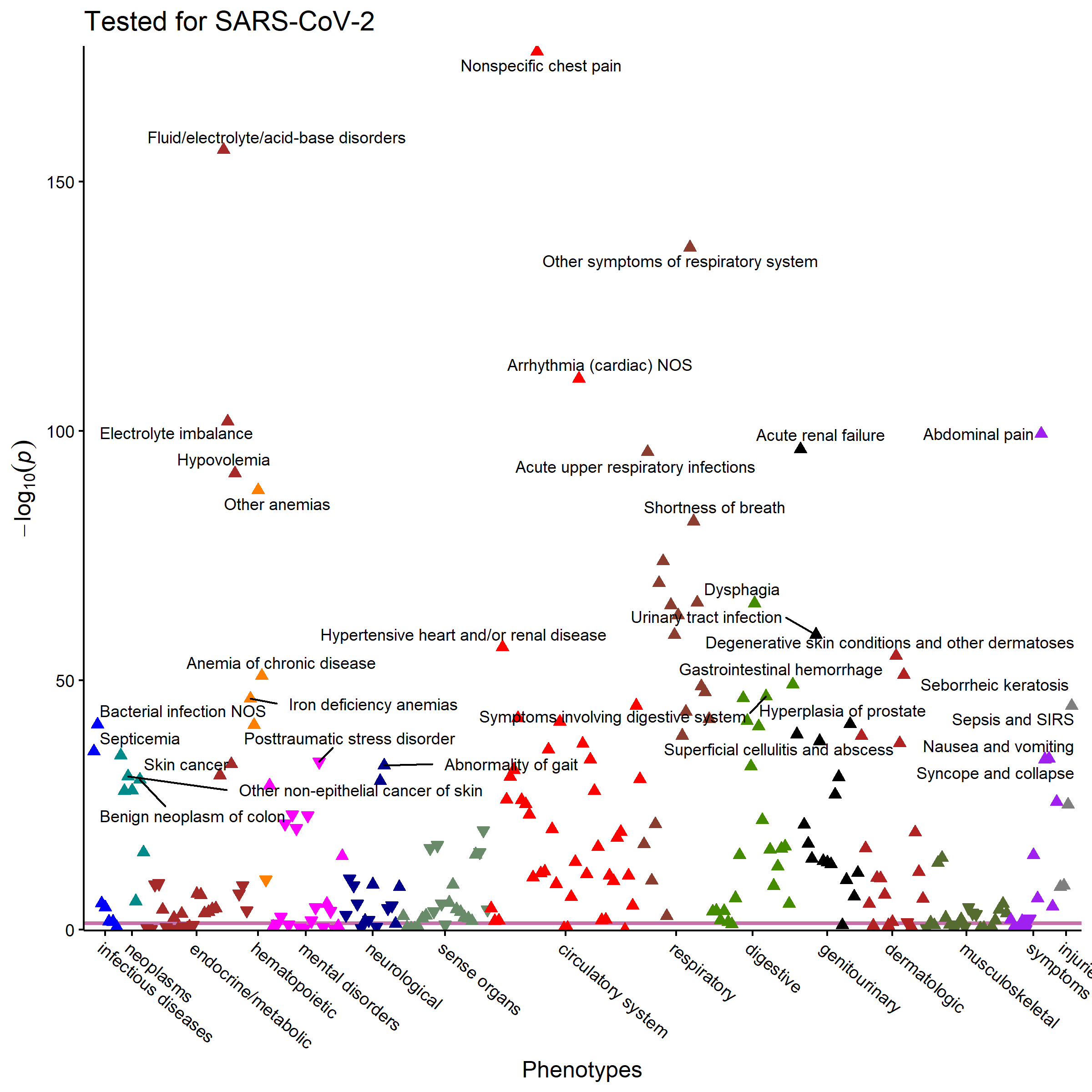

Supplement: S2 Fig — (TIFF) [file pone.0251651.s003.tiff]

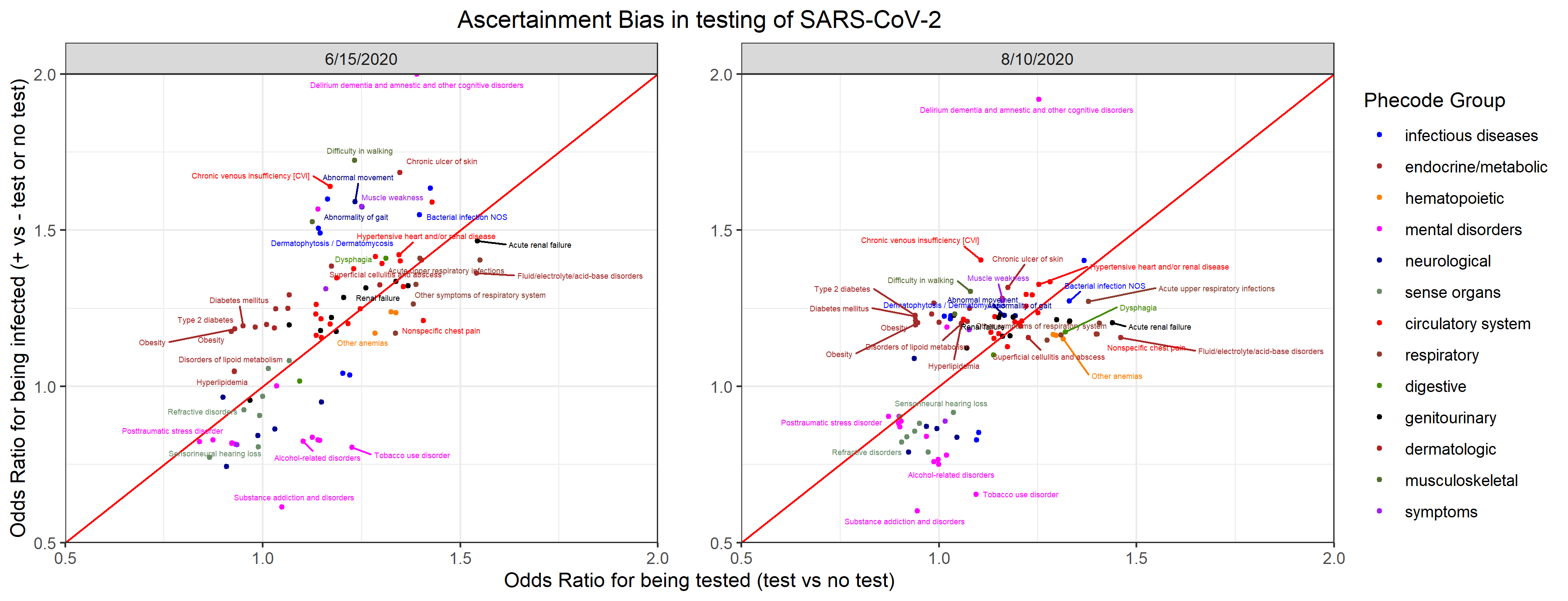

Supplement: S3 Fig — (TIFF) [file pone.0251651.s004.tiff]
